# The Role of Non-Coding RNAs in the Pathogenesis of Asthma

**DOI:** 10.3390/genes17101182

**Published:** 2026-09-24

**Authors:** Radosław Birger, Paulina Plewa, Nina Komaniecka, Estera Bakinowska, Patryk Graczyk, Andrzej Pawlik

**Affiliations:** 1 Department of Experimental and Clinical Pharmacology, Pomeranian Medical University, 70-111 Szczecin, Poland; radoslaw.birger@pum.edu.pl (R.B.); nina.komaniecka@pum.edu.pl (N.K.); 2 Laboratory of Pharmacodynamics, Pomeranian Medical University, 71-244 Szczecin, Poland; 3 Department of Physiology, Pomeranian Medical University, 70-111 Szczecin, Poland; paulina.plewa@op.pl (P.P.); esterabakinowska@gmail.com (E.B.); 4 University Clinical Hospital in Poznan, 60-355 Poznan, Poland; patryk.graczyk@usk.poznan.pl

**Keywords:** Asthma, non-coding RNAs, microRNAs, long non-coding RNAs, circular RNAs, airway inflammation, airway remodeling, biomarkers

## Abstract

Asthma is a chronic inflammatory airway disease characterized by considerable clinical and molecular heterogeneity, involving complex interactions between genetic susceptibility, environmental factors, and immune dysregulation. Increasing evidence indicates that non-coding RNAs (ncRNAs), including microRNAs (miRNAs), long non-coding RNAs (lncRNAs), circular RNAs (circRNAs), and other regulatory RNA species, play essential roles in the development and progression of asthma—modulate inflammatory responses, immune cell differentiation, airway remodeling, and epithelial barrier function by interacting with key molecular and signaling pathways, notably the NF-κB, JAK/STAT, PI3K/AKT/mTOR, and TGF-β/Smad cascades. For example, miR-125b-5p and miR-223-3p demonstrate consistent dysregulation across varying biological compartments and independent cohorts, while miR-21, miR-146a, miR-155, and lncRNAs like NEAT1 and MALAT1 are frequently confirmed as central regulators of airway pathology. Aberrant expression of these ncRNAs has been associated with distinct asthma phenotypes, disease severity, and therapeutic responses, highlighting their potential as diagnostic and prognostic biomarkers. This review summarizes current knowledge on the biological functions of ncRNAs in asthma, their involvement in critical pathogenic mechanisms, and discusses their emerging clinical applications and the challenges of translating RNA-based therapeutics into clinical practice.

## 1. Introduction

Asthma is one of the most common chronic non-communicable diseases worldwide, defined clinically by respiratory symptoms—wheeze, breathlessness, chest tightness, and cough—together with variable expiratory airflow limitation, and it remains a major and growing global health burden [1,2].

Asthma also remains inadequately treated: in a global management survey, more than half of adults and adolescents with severe symptoms were not receiving inhaled corticosteroids [3].

Asthma is clinically and biologically heterogeneous. Genetic predisposition and environmental exposures interact throughout life, shaping an individual’s risk of disease. Prevention strategies aim to reduce these risks [4,5]. Many patients show type 2 airway inflammation, characterised by eosinophilia, elevated immunoglobulin E (IgE) and corticosteroid responsiveness. Others lack demonstrable type 2 inflammation and respond poorly to therapies directed against it [6,7].

The pathogenesis of asthma is conventionally described as a disordered interaction between the airway epithelium, the immune system, and the structural cells of the airway wall [8,9]. Injury to the epithelium by allergens, viruses, pollutants, and irritants initiates immune activation. Most patients present a type 2 response, although other inflammatory mechanisms operate in a substantial minority of patients [6]. Persistent inflammation is accompanied by airway remodelling: subepithelial fibrosis, smooth muscle hyperplasia, epithelial-to-mesenchymal transition (EMT), and mucous gland hyperplasia [10]. Remodelling contributes to airway hyperresponsiveness and to the incompletely reversible airflow limitation of long-standing disease [10].

ncRNAsare attracting growing interest as regulators of these processes in asthma. Genome-scale transcriptome mapping has established that most of the human genome is transcribed and that the majority of transcripts do not encode protein [11,12]. Whether all such transcription is functional remains debated, given that biochemical activity is not synonymous with biological function [13]. A growing subset of ncRNAs has nonetheless been assigned specific regulatory roles. They guide silencing complexes to complementary transcripts, scaffold chromatin-modifying enzymes at genomic loci, act as decoys that sequester RNA-binding proteins and other RNAs, and direct site-specific modification of other RNA species [14,15]. ncRNA expression is markedly cell type- and state-specific, so ncRNA profiles indicate which airway cells are active and in which state [14,16]. ncRNAs can also be targeted directly with synthetic oligonucleotides, an approach that has already reached clinical development in other diseases [17,18].

## 2. Pathogenesis of Asthma

Asthma is a complex, heterogeneous disease arising from the interplay between genetic susceptibility and environmental exposures (Figure 1).

Genetic factors contribute to disease susceptibility, with genome-wide association studies identifying multiple asthma-associated loci, including IL33, IL1RL1/IL18R1, HLA-DQ, SMAD3, and IL2RB, as well as the 17q21 region encompassing ZPBP2, GSDMB, and ORMDL3 [19,20,21,22]. These genetic factors interact with environmental exposures, including allergens, secondhand tobacco smoke, and indoor and ambient air pollution, which may influence disease onset, severity, and phenotype [23,24,25,26]. Environmental factors can induce airway epithelial injury and oxidative stress, impair epithelial barrier integrity, and promote the release of inflammatory mediators, thereby facilitating allergen penetration and persistent airway inflammation [27,28,29]. Asthma is characterized by heterogeneous inflammatory and immune responses, including type 2–high eosinophilic inflammation driven by Th2-associated cytokines such as IL-4, IL-5, and IL-13, as well as non-type 2 responses involving Th1-, Th17-, and innate immune pathways, which may be associated with neutrophilic inflammation [30,31,32,33]. Persistent inflammation, together with epithelial injury and altered airway smooth muscle function, contributes to airway hyperresponsiveness and structural changes collectively referred to as airway remodeling, including goblet cell hyperplasia, increased airway smooth muscle mass, basement membrane thickening, and subepithelial fibrosis [34,35,36,37,38]. Thus, the interaction between genetic predisposition, environmental exposures, epithelial dysfunction, oxidative stress, immune dysregulation, and structural airway changes contributes to the heterogeneous clinical phenotypes and progression of asthma.

## 3. Dysregulation of ncRNAs in Asthma

nc RNAs are transcribed but not translated into protein and are conventionally grouped by length into small species (<200 nt) and long species (>200 nt); circular RNAs form a further, topologically distinct class defined by a covalently closed structure rather than by size [13]. The best-characterised classes—miRNAs, lncRNAs and circRNAs—together with several smaller classes still under active characterisation (Figure 2), regulate gene expression through diverse post-transcriptional and epigenetic mechanisms, including silencing-complex guidance, scaffolding of chromatin-modifying enzymes, competitive sequestration of RNA-binding proteins and other RNAs (“sponging”), and site-specific RNA modification [11,12]. Because their expression is markedly cell-type and state-specific, ncRNAs are increasingly implicated in the pathophysiology of a wide range of diseases, including asthma, and their asthma-specific dysregulation and mechanistic roles are discussed below [12,14].

Accumulating transcriptomic evidence indicates that asthma is accompanied by extensive dysregulation of ncRNA expression. Rather than forming a single and uniform disease-associated pattern, ncRNA profiles appear to vary according to the biological compartment analysed, cellular origin, RNA subclass, treatment exposure, and clinical characteristics of the studied population. This heterogeneity is particularly relevant in asthma, where distinct inflammatory phenotypes and molecular endotypes may coexist under the same clinical diagnosis. Consequently, ncRNA profiles derived from peripheral blood cannot be assumed to directly reproduce those detected in airway-derived samples, and apparently divergent results between studies may reflect biological and methodological differences rather than true inconsistency [39,40,41,42,43,44,45,46,47,48,49,50,51].

Broad changes in lncRNA expression have been demonstrated in circulating cells of patients with asthma. Ma et al. [39] identified 1137 differentially expressed lncRNAs in peripheral blood from patients with asthma compared with healthy controls, including 485 upregulated and 652 downregulated transcripts, and subsequently confirmed selected candidates by reverse transcription–quantitative polymerase chain reaction (RT-qPCR). In children with asthma, Xia et al. [40] identified 719 differentially expressed lncRNAs in peripheral blood mononuclear cells (PBMCs) compared with age-matched controls. Among them, lnc-BAZ2B was predominantly expressed in monocytes and was further subjected to functional characterisation. These findings indicate that asthma-associated transcriptional changes involve a broad lncRNA repertoire rather than a small number of isolated transcripts.

Recent studies have extended these observations to circulating miRNAs. Luo et al. [41] used high-throughput sequencing to investigate miRNA expression in PBMCs from children with asthma and subsequently validated selected candidates, including altered expression of miR-31-5p. Importantly, not all signals obtained during the initial sequencing stage were reproduced during subsequent validation, highlighting the importance of independent confirmation of high-dimensional ncRNA datasets. A larger whole-blood small RNA sequencing analysis by Tiwari et al. [42] included 361 children with asthma and identified two miRNAs associated with airflow below a predefined threshold. The authors reported an increase in Let-7e-5p and a decrease in miR-342-3p in children with lower airflow, with concordant directions of association observed in an independent adult cohort with airflow obstruction. These results suggest that circulating ncRNA patterns may reflect specific physiological features of disease rather than asthma status alone.

Airway-derived samples provide additional evidence for marked compartment specificity. In bronchial biopsies, Roffel et al. [43] identified 78 differentially expressed miRNAs in patients with asthma compared with healthy controls, of which 60 remained significantly different after adjustment for smoking and inhaled corticosteroid treatment. Several of these miRNAs were also associated with clinical and inflammatory characteristics of asthma, with miR-125b-5p and miR-223-3p emerging as particularly relevant candidates. Bronchial ncRNA expression may also vary according to disease course. Boudewijn et al. [44] compared bronchial biopsies from individuals with complete asthma remission, persistent asthma, and healthy controls. They identified distinct miRNA expression patterns associated with remission and, through integrated network analysis, demonstrated that both miRNAs and lncRNAs are prominent components of the molecular network characterising complete remission. Thus, bronchial ncRNA signatures may reflect not only the presence of asthma but also specific longitudinal disease states.

Differences between systemic and pulmonary compartments have also been observed within severe asthma. Gil-Martínez et al. [45] analysed miRNA profiles in serum and lung tissue from individuals with severe asthma according to oral corticosteroid (OCS) exposure. Next-generation sequencing identified 11 differentially expressed serum miRNAs, with five subsequently validated, while a different set of miRNAs was altered in lung tissue. These observations should primarily be interpreted as treatment-associated differences within severe asthma rather than as a direct comparison between asthma and health. Nevertheless, the non-identical profiles detected in serum and lung tissue emphasise that treatment exposure and biological compartment can substantially influence ncRNA expression.

Compartment specificity is similarly evident for extracellular vesicle (EV)-associated ncRNAs. Khalyfa et al. [46] compared exosomal ncRNA profiles in plasma and saliva from children with asthma stratified by lung function. They identified 27 differentially expressed miRNAs in plasma and 40 in saliva between children with severe airflow obstruction and those with normal lung function, while only five differentially expressed miRNAs were shared between both compartments. More directly relevant to airway inflammation, Sanz-Rubio et al. [47] performed small RNA sequencing of exosomal RNA from plasma and induced sputum in healthy individuals and in patients with severe T2-high or T2-low asthma. Each inflammatory endotype showed distinct expression profiles in both plasma and induced sputum, and selected miRNAs were subsequently validated in an independent cohort. These findings demonstrate that both sampling compartment and inflammatory endotype contribute to the ncRNA signature identified in severe asthma.

Bronchial epithelial profiling has further expanded the spectrum of ncRNAs implicated in asthma beyond miRNAs and lncRNAs. In 2026, Zhou et al. [48] integrated transfer RNA-derived small RNA (tsRNA) and mRNA profiles from bronchial epithelial cells of patients with severe asthma and healthy controls. The analysis identified 58 dysregulated tsRNAs and 961 differentially expressed mRNAs and generated a regulatory network comprising 343 negatively correlated tsRNA-mRNA pairs. The authors validated 36 hub mRNAs in an independent dataset and assessed selected tsRNAs in HDM-stimulated bronchial epithelial cells. These results support the concept that emerging classes of ncRNAs may contribute to the molecular heterogeneity of the asthmatic airway and demonstrate the value of studying disease-relevant structural cells directly.

ncRNA signatures have also been associated with asthma severity and specific inflammatory phenotypes. Vorobeva et al. [49] analysed 35 candidate serum miRNAs in individuals without asthma and in patients with mild or severe asthma [11]. Compared with mild asthma, severe asthma is characterised by increased miR-223-3p together with decreased miR-30a-5p, miR-660-5p, and miR-125b-5p [49]. In addition, individual miRNAs are associated with clinical indices of more severe disease and airway inflammation. Phenotype-associated ncRNA variation is not limited to miRNAs. Li et al. [50] demonstrated that circulating piRNAs are associated with eosinophil counts and total serum IgE concentrations in children with asthma and identified a piRNA associated with T2-high asthma phenotypes. Alongside the airflow-associated blood miRNAs reported by Tiwari et al. [42] and the T2-high and T2-low exosomal profiles reported by Sanz-Rubio et al. [47], these observations indicate that ncRNA signatures may capture distinct biological dimensions of asthma, including airflow impairment, type 2 inflammation, and disease severity.

Bronchoalveolar lavage fluid (BALF) represents an attractive matrix for the investigation of airway ncRNAs because compared with peripheral blood, it provides a more direct sample of the lower respiratory tract. However, there are markedly fewer contemporary human asthma-specific ncRNA datasets based on BALF relative to those derived from blood, induced sputum, bronchial biopsies, or bronchial epithelial cells. Recent methodological reviews have also emphasised substantial differences among airway-derived biosamples in terms of cellular composition, RNA content, collection procedures, and pre-analytical processing [51]. Therefore, current tissue-specific ncRNA comparisons should consider the relative scarcity of recent BALF data rather than assuming that all airway compartments have been characterised to a similar extent.

Overall, the available evidence indicates that ncRNA dysregulation in asthma is extensive but strongly context-dependent. Whole blood, PBMCs, serum, EVs, induced sputum, bronchial tissue, and bronchial epithelial cells provide overlapping but non-identical molecular profiles [39,40,41,42,43,44,45,46,47,48,49,50]. Disease severity, airflow limitation, inflammatory endotype, treatment exposure, and disease course introduce additional sources of biological variation. Accordingly, discrepancies between reported ncRNA signatures should be interpreted in light of differences in cohort composition, sampling compartment, RNA fraction, analytical platform, normalisation strategy, and validation methodology. Reproducible characterisation of asthma-associated ncRNA profiles will require independent validation in well-phenotyped cohorts and, where feasible, parallel analysis of systemic and airway-derived samples.

This heterogeneity is itself an important observation: rather than pointing to a small, consistently replicated set of transcripts, the published evidence supports a broad and compartment-dependent ncRNA response to asthma. A systematic comparison of the studies above (Table 1) shows that direct cross-study overlap is more evident for miRNAs than for lncRNAs: miR-125b-5p and miR-223-3p are independently reported as altered in bronchial biopsies and in serum, in studies differing in compartment, cohort and disease severity, and thus represent stronger candidate biomarkers than signals reported by a single study alone. Among lncRNAs, the two large blood/PBMC screens together implicate well over a thousand differentially expressed transcripts, but only one candidate (lnc-BAZ2B) has so far been carried forward for independent functional characterisation; the two datasets have not yet been directly cross-compared at the level of individual loci. A formal re-analysis and cross-comparison of the underlying public transcriptomic datasets—including the GSE195599 dataset generated by Ma et al.—against other publicly available asthma transcriptomic datasets was beyond the scope of the present narrative review, but represents a concrete and valuable next step for identifying which additional lncRNA signals replicate across independent cohorts and for distinguishing robust candidate biomarkers from study-specific findings.

## 4. Role of miRNAs in Asthma Pathogenesis

### 4.1. Regulation of Innate and Adaptive Immune Responses

Various miRNAs regulate both innate and adaptive immune responses in asthma by modulating Toll-like receptor (TLR) signalling, cytokine production, and T cell differentiation. In particular, miR-146 negatively regulates TLR4 signalling by targeting interleukin-1 receptor-associated kinase 1 (IRAK1) and TNF receptor-associated factor 6 (TRAF6), whereas miR-21, miR-126, miR-145, and miR-221 influence Th2 cytokine production and allergic airway inflammation, highlighting the important role of miRNAs in controlling immune homeostasis during asthma [63].

Differentially expressed circulating miRNAs in asthma may regulate multiple inflammatory pathways, including mitogen-activated protein kinase (MAPK), NF-κB, phosphoinositide 3-kinase (PI3K)–AKT and signal transducer and activator and transcription (STAT)-mediated signalling [64]. In particular, miR-146a may act as a negative-feedback regulator of inflammation by suppressing NF-κB activation and cytokine signalling [65].

miRNAs can regulate airway inflammation through complex interactions with transcriptional and post-transcriptional mechanisms. Another example is miR-570-3p, which directly targets the RNA-binding protein Hu antigen R (HuR), a central regulator of epithelial-derived cytokines, chemokines and other inflammatory mediators. Modulation of miR-570-3p levels in human airway epithelial cells alters the expression of multiple inflammatory mediators, including IL-6, C-C motif chemokine ligand 2 (CCL2), CCL4, CCL5, and IL-8. Interestingly, miR-570-3p increases the expression of some inflammatory mediators while suppressing TNF-α-induced expression of others, indicating that its effects depend on the cellular and inflammatory context. Computational analyses further suggest potential involvement of STAT1, STAT3, and sirtuin 1 (SIRT1)/NF-κB signalling in these effects. These findings highlight the complexity of miRNA-mediated regulation of airway inflammation, in which both direct targeting and indirect modulation of signalling pathways may contribute to the final inflammatory response [66].

Interestingly, the effects of miRNAs on airway inflammation cannot always be predicted from their canonical function as post-transcriptional repressors. miR-570-3p, for example, simultaneously increases and suppresses the expression of different inflammatory mediators, suggesting that its effects may depend on indirect interactions with transcription factors, RNA-binding proteins, and signalling pathways. Such complex regulatory networks highlight the context-dependent nature of miRNA activity in asthma [66].

### 4.2. Th1/Th2/Th17/Treg Balance

A range of miRNAs contribute to the regulation of Th cell polarisation in asthma. miR-21 promotes Th2 differentiation by suppressing IL-12 expression, thereby shifting the immune response towards a Th2 phenotype [67,68]. miR-155 is involved in T cell differentiation and can influence both Th1- and Th2-associated responses in a context-dependent manner [69,70,71]. In addition, miR-126 and the let-7 family regulate Th2 immune responses through modulation of IL-5 and IL-13 expression [63,72]. Moreover, miR-570-3p may influence adaptive immune responses indirectly through the regulation of epithelial inflammatory mediators and signalling molecules [66]. These findings demonstrate the important role of miRNAs in maintaining the Th1/Th2 balance. The Th17/T regulatory cell (Treg) axis may also be regulated by miRNAs, although evidence in asthma is considerably more limited than for Th2-associated responses. miR-155 appears to be one of the best-characterised regulators of this balance. In a murine model of asthma, modulation of miR-155 alters the Th17/Treg balance through differential activation of STAT3 and STAT5, with increased miR-155 favouring Th17 responses and reduced miR-155 associated with enhanced Treg responses [73]. Mechanistically, miR-155 can promote Th17 differentiation and IL-17A production by targeting suppressor of cytokine signalling 1 (SOCS1), thereby enhancing Janus Kinase (JAK)/STAT signalling [74]. miR-155 also contributes to Th17 cell function through suppression of Jarid2, relieving polycomb-mediated transcriptional repression of Th17-associated genes [75]. These findings suggest that miRNA-dependent regulation of the Th17/Treg axis may contribute to the inflammatory imbalance observed in asthma, particularly in phenotypes characterised by Th17-associated and neutrophilic inflammation.

### 4.3. Eosinophilic and Neutrophilic Inflammation

Multiple miRNAs contribute to eosinophilic airway inflammation by regulating key Th2 cytokines. Panganiban et al. [76] demonstrated that miR-1248 regulates IL-5, a key Th2 cytokine responsible for eosinophil survival, differentiation, recruitment, and activation. Interestingly, unlike the classical inhibitory function of miRNAs, miR-1248 enhanced IL-5 expression through interaction with its 3′-UTR, suggesting a potential pathogenic role in promoting eosinophilic inflammation. Increased expression of miR-1248 in asthma suggests that this miRNA may contribute to enhanced Th2 responses and therefore represents a potential therapeutic target.

There is an association between a circulating miRNA profile characterised by increased miR-126, miR-21, and let-7b and the eosinophilic asthma phenotype [68,77]. Among these miRNAs, miR-21 is particularly strongly increased, with more than an eightfold elevation in the eosinophilic asthma cluster, and it promotes eosinophil precursor growth and enhances eosinophilia [64]. miR-223 may also contribute to eosinophilic inflammation by regulating eosinophil progenitor proliferation [78].

Inhibition of miR-126 reduces airway hyperresponsiveness, mucus production, eosinophil and neutrophil recruitment, and the expression of IL-5 and IL-13 [72,79]. Similarly, suppression of miR-145 decreases goblet cell hyperplasia, peribronchial eosinophilia, and Th2 cytokine production [80]. Knockdown of miR-106a also alleviates airway inflammation, reduces goblet cell metaplasia and subepithelial fibrosis, and enhances IL-10 expression [81]. There is limited evidence regarding the regulation of neutrophilic inflammation, although inhibition of miR-126 also reduces neutrophil recruitment [72].

Dysregulation of specific miRNAs may contribute to neutrophilic airway inflammation in asthma. In particular, miR-223-3p is a key component of a sputum miRNA network associated with increased airway neutrophilia and severe asthma. miR-223-3p is highly expressed in airway neutrophils and is associated with gene networks involved in TLR signalling, IL-17 responses, and endoplasmic reticulum stress, suggesting that it may contribute to the regulation of neutrophil-associated inflammatory responses. Other miRNAs within the same network, including miR-15b-5p, miR-25-3p and members of the let-7 family, have also been linked to neutrophilic inflammation and/or Th17-related responses [82].

### 4.4. Airway Epithelial Dysfunction

miRNAs also contribute to airway epithelial homeostasis. In particular, miR-146 has been shown to inhibit cytokine-induced apoptosis in human bronchial epithelial cells, suggesting a protective role in maintaining epithelial integrity [63].

Interestingly, miR-146a and let-7 exhibit compartment-specific expression patterns in asthma, with increased levels detected in the circulation but decreased levels reported in lung-derived samples, including airway epithelial cells. This difference may reflect impaired miRNA transfer between the circulation and the lungs and/or reduced pulmonary expression of anti-inflammatory miRNAs [76,83].

The airway epithelium is an important source of IL-1α and IL-1β following exposure to allergens or inhaled irritants. IL-1 signalling in epithelial cells can stimulate the production of inflammatory mediators, including IL-6, granulocyte-macrophage colony-stimulating factor (GM-CSF), IL-33, and thymic stromal lymphopoietin (TSLP), thereby promoting airway inflammation and allergic sensitisation. These findings suggest that dysregulation of miRNA-dependent inflammatory pathways may contribute to the altered communication between airway epithelial cells and immune cells characteristic of asthma [84]. Hence, dysregulation of miRNAs may contribute not only to airway inflammation but also to epithelial dysfunction observed in asthma.

miR-570-3p has been specifically investigated in human airway epithelial cells and found to regulate the RNA-binding protein HuR, a key post-transcriptional regulator of epithelial-derived inflammatory mediators. Altering miR-570-3p expression affects the production of several cytokines and chemokines, including IL-6, CCL2, CCL4, CCL5, and IL-8. These effects are not uniformly inhibitory, suggesting that miR-570-3p can regulate epithelial inflammatory responses through both direct and indirect mechanisms. Notably, miR-570-3p levels are significantly reduced in exhaled breath condensate from patients with asthma compared to healthy controls. Together, these findings suggest that dysregulation of miR-570-3p may contribute to abnormal inflammatory signalling within the airway epithelium and may represent a mechanism linking altered miRNA expression with persistent airway inflammation in asthma [66].

### 4.5. Airway Remodeling and Fibrosis

Specific miRNAs have been implicated in airway remodelling by regulating structural changes within the airways. Administration of let-7 mimics reduces mucus metaplasia and subepithelial fibrosis while attenuating airway inflammation and hyperresponsiveness through inhibition of IL-13 expression [77]. Likewise, knockdown of miR-106a alleviates goblet cell metaplasia and subepithelial fibrosis by restoring IL-10 expression [81]. Furthermore, inhibition of miR-145 reduces goblet cell hyperplasia, whereas miR-133a regulates airway smooth muscle contractility through suppression of RhoA expression, suggesting a role in airway hyperresponsiveness and remodelling [80,85].

Several differentially expressed miRNAs are predicted to target different isoforms of the TGF-β receptor, suggesting a potential role in airway remodelling. In addition, miRNAs targeting focal adhesion pathways may contribute to structural changes involving the airway basement membrane and smooth muscle [64]. IL-1 signalling may further contribute to these processes through its effects on lung fibroblasts and ECM regulation. Airway epithelial–derived IL-1α has been shown to modulate fibroblast production of inflammatory cytokines and ECM proteins and to interact with the profibrotic TGF-β pathway. Given that miR-146a-5p can suppress IL-1 signalling by targeting IRAK1, this miRNA may potentially influence inflammatory processes linked to tissue repair and remodelling, although a direct role of miR-146a in airway remodelling remains to be established [84].

miRNAs have been implicated in the regulation of airway remodelling and fibrosis in asthma by modulating signalling pathways involved in airway smooth muscle activation, fibroblast function, and ECM deposition. Among the best-characterised miRNAs in this context is miR-21, which has been shown to promote airway remodelling through regulation of the TGF-β/Smad pathway. TGF-β1 induces miR-21 expression in human bronchial fibroblasts, while miR-21 targets the inhibitory regulator Smad7 and TGFβRII. In particular, increased miR-21 expression under pathological conditions reduces Smad7 protein levels, thereby attenuating the negative regulation of TGF-β/Smad signalling and favouring profibrotic responses. This is accompanied by increased expression of collagen, alpha-smooth muscle actin (α-SMA), and fibronectin, which contribute to ECM deposition and structural remodelling of the airway. Consistently, inhibition of miR-21 attenuates airway remodelling and reduces collagen and other profibrotic gene expression in bronchial smooth muscle cells and experimental models of chronic allergic asthma. These findings indicate that miR-21 acts predominantly as a profibrotic and pro-remodelling miRNA in asthma, with the miR-21–TGF-β–Smad7 axis representing an important regulatory pathway underlying airway structural changes [86,87].

In contrast, miR-133a appears to exert an anti-remodelling effect in asthma. Its expression is reduced in asthmatic airways, whereas miR-133a overexpression attenuates airway remodelling in experimental models. miR-133a directly targets the 3′-UTR of the insulin-like growth factor 1 receptor (IGF1R), resulting in inhibition of downstream PI3K/AKT/mechanistic target of rapamycin (mTOR) signalling. Increased miR-133a expression consequently reduces the activation of airway smooth muscle cells and the expression of α-SMA, while its inhibition enhances these remodelling-associated changes. In vivo, restoration of miR-133a expression alleviates airway wall thickening and airway smooth muscle hypertrophy, whereas suppression of miR-133a aggravates these features. Thus, the miR-133a–IGF1R–PI3K/AKT/mTOR axis represents a regulatory mechanism through which miRNA dysregulation may contribute to airway smooth muscle remodelling [88].

Other miRNAs may regulate airway fibrosis indirectly through modulation of cytokine signalling. Members of the let-7 family directly target the 3′-UTR of IL-13 and suppress its expression. In experimental allergic asthma, administration of let-7 reduces IL-13 expression and is associated with attenuation of subepithelial fibrosis, suggesting an anti-fibrotic effect of let-7-mediated regulation. In contrast to miR-21 and miR-133a, however, the available evidence indicates that the effect of let-7 on airway fibrosis is mediated primarily through suppression of IL-13 rather than through direct regulation of a core fibrotic pathway. miR-155-5p has also been implicated in airway remodelling, as its overexpression promotes proliferation and migration of bronchial smooth muscle cells following IL-13 stimulation, suggesting a potential pro-remodelling role. Conversely, the role of miR-126 appears to be context-dependent, as its inhibition attenuates asthmatic features in a model of acute allergic asthma but does not significantly affect airway inflammation or remodelling in a model of chronic allergic asthma. Collectively, these findings indicate that miRNAs can exert both pro- and anti-remodelling effects in asthma, with their functional impact depending on the specific miRNA, cellular target, signalling pathway, and disease context [77].

### 4.6. Key Asthma-Associated miRNAs

A number of miRNAs have emerged as important regulators of asthma pathogenesis, acting through diverse molecular pathways that influence airway inflammation, immune cell differentiation, epithelial function, and structural remodeling (Table 2). Their effects are highly context-dependent and may be either pro-inflammatory or protective, depending on the specific miRNA, its cellular source, and the anatomical compartment in which it is expressed.

The miRNAs summarized in Table 2 illustrate several major mechanisms through which miRNA dysregulation may contribute to asthma. These include modulation of cytokine signaling and T-cell differentiation, regulation of innate immune pathways such as TLR/NF-κB signaling, and the control of pathways involved in airway smooth muscle activation and extracellular matrix remodeling. Importantly, several miRNAs appear to influence multiple components of asthma pathogenesis rather than acting within a single inflammatory pathway. miR-145 has been associated with eosinophilic inflammation and airway remodeling, including goblet cell hyperplasia and peribronchial eosinophilia [63,80]. miR-221 has been implicated in the regulation of allergic airway inflammation and mast cell activation, whereas miR-25 has been associated with inflammatory responses and airway smooth muscle phenotype [63,82]. miR-570-3p represents an additional example of a context-dependent regulator of airway inflammation. By targeting the RNA-binding protein HuR in airway epithelial cells, miR-570-3p can modulate the production of several epithelial-derived cytokines and chemokines, although its effects are not uniformly inhibitory. Reduced miR-570-3p levels have also been detected in exhaled breath condensate from patients with asthma, suggesting a potential association with persistent airway inflammation [66]. Other miRNAs, including miR-1291, have been identified as differentially expressed in asthma and may participate in the regulation of Th2-associated responses, although their functional significance remains less well characterized [76].

The biological effects of individual miRNAs may also vary according to their cellular and anatomical compartment. This is particularly evident for miR-146a, for which increased circulating levels have been reported together with reduced expression in airway epithelial and bronchial tissue. Such compartment-dependent regulation suggests that circulating miRNA levels may not necessarily reflect local pulmonary expression or activity [96]. A similar compartment-dependent variation has been reported for miR-155, further emphasizing the importance of considering the biological source of miRNAs when interpreting their association with asthma [94,97].

Collectively, these findings indicate that miRNA dysregulation in asthma represents a complex and interconnected regulatory network involving both disease-promoting and protective mechanisms. Rather than acting as isolated regulators, individual miRNAs may simultaneously affect several cellular processes and signaling pathways, while different miRNAs may converge on common inflammatory or remodeling pathways. This network-based regulation may partly explain the heterogeneity of miRNA expression patterns observed across asthma phenotypes and biological compartments.

Despite increasing evidence for their involvement in asthma pathogenesis, the functional significance of several asthma-associated miRNAs remains incompletely defined. In particular, although miR-34a has been implicated in the regulation of HuR and epithelial-derived inflammatory mediators, its specific contribution to asthma pathogenesis remains to be established [95,96]. Similarly, the functional roles of several additional differentially expressed miRNAs require further investigation to determine whether their altered expression represents a causal mechanism, a consequence of airway inflammation or a secondary reflection of changes in cellular composition.

## 5. Role of lncRNAs in Asthma

lncRNAs are increasingly recognised as functional regulators of cellular processes involved in asthma. Experimental studies indicate that selected lncRNAs can influence inflammatory signalling, immune cell differentiation and activation, airway smooth muscle cell behaviour and EMT. Their mechanisms include modulation of transcription, interaction with genomic regulatory regions, regulation of RNA stability, competition with miRNAs, and intercellular transfer within EVs. However, the strength of evidence varies considerably among individual lncRNAs. Some candidates are supported by observations in human samples combined with mechanistic experiments, whereas others have primarily been characterised in cultured cells or experimental models of allergic asthma [40,99,100,101,102,103,104,105].

Xia et al. [40] reported a direct connection between immune cell activation and lnc-BAZ2B. identified lnc-BAZ2B among the differentially expressed lncRNAs in PBMCs of children with asthma and demonstrated that it was predominantly expressed in monocytes. Mechanistically, lnc-BAZ2B stabilises BAZ2B pre-mRNA, thereby increasing expression of BAZ2B. BAZ2B subsequently enhances transcription of IFN regulatory factor 4 and promotes alternative M2 macrophage activation. Functional experiments supported the relevance of this pathway in asthma-related inflammation, providing an example in which a lncRNA identified in human paediatric samples was linked to a defined immune-regulatory mechanism.

There is evidence that lncRNAs participate in the regulation of adaptive immune cell differentiation. Ji et al. [99] demonstrated increased LincR-PPP2R5C expression in CD4+ T cells from asthmatic mice and during Th2 differentiation [99]. LincR-PPP2R5C interacts with the PPP2R5C promoter by forming an RNA–DNA triplex, increasing PPP2R5C expression, and modulating protein phosphatase 2A (PP2A) activity. Global and CD4+ T cell–specific deletion of LincR-PPP2R5C reduces Th2 differentiation, airway hyperresponsiveness, and inflammatory responses in an acute experimental model of allergic asthma [99]. These findings demonstrate that lncRNAs can influence immune cell fate through direct interaction with genomic regulatory regions rather than exclusively through post-transcriptional RNA–RNA interactions.

lncRNAs also participate in inflammatory signalling between immune and structural airway cells. Colorectal neoplasia differentially expressed (CRNDE) provides a well-characterised example of this mechanism. In one study, researchers transferred CRNDE from activated neutrophils to airway smooth muscle cells within exosomes [100]. They found that neutrophil-derived exosomal CRNDE increases airway smooth muscle cell proliferation and migration, and activates NF-κB signalling through enhanced phosphorylation of inhibitor of nuclear factor kappa-B kinase beta (IKKβ). In an in vivo experimental model of asthma, knockdown of neutrophil CRNDE reduces bronchial smooth muscle thickness. This mechanism connects neutrophilic inflammation with structural airway changes and illustrates how lncRNAs can function as mediators of intercellular communication during airway remodelling.

Metastasis-associated lung adenocarcinoma transcript 1 (MALAT1) has also been implicated in abnormal airway smooth muscle responses. Huang et al. [101] demonstrated that MALAT1 promotes proliferation and migration of airway smooth muscle cells in an asthma-related experimental system, whereas its suppression attenuates these effects through regulation of miR-216a. In a subsequent study, Chiba et al. [102] identified an additional mechanism involving bronchial smooth muscle contractility. In cultured human bronchial smooth muscle cells, inhibition of MALAT1 prevents IL-13-induced upregulation of RhoA protein. An miR-133a-3p mimic produces a similar effect, while inhibition of miR-133a-3p reverses the effect of MALAT1 suppression. In bronchial smooth muscle from an experimental murine model of asthma, increased MALAT1 and RhoA expression is accompanied by a decrease in miR-133a-3p. Taken together, the available MALAT1 may influence both proliferative responses and the contractile phenotype of airway smooth muscle through distinct miRNA-dependent mechanisms.

Maternally expressed gene 3 (MEG3) has also been implicated in airway smooth muscle cell regulation. Gu and Zhou [103] investigated MEG3 in human airway smooth muscle cells stimulated with platelet-derived growth factor-BB (PDGF-BB). MEG3 knockdown reduces proliferation and migration, while mechanistic experiments implicated a regulatory relationship involving miR-143-3p and fibroblast growth factor 9 (FGF9). miR-143-3p directly targets FGF9, and restoration experiments support the contribution of this axis to the observed changes in cell proliferation and migration. These findings indicate that MEG3 can modulate remodelling-associated airway smooth muscle cell behaviour. However, this evidence is mostly from an in vitro model, and the authors emphasised the need for in vivo validation and direct assessment of the MEG3/miR-143-3p/FGF9 axis in patients with asthma [103].

Airway epithelial plasticity represents another important target of lncRNA regulation. Nuclear paraspeckle assembly transcript 1 (NEAT1) has been investigated in the context of EMT, a process implicated in structural airway remodelling. Li et al. [104] used TGF-β1 to induce EMT in BEAS-2B bronchial epithelial cells and observed increased NEAT1 expression. Knockdown of NEAT1 decreases the mesenchymal markers N-cadherin, vimentin, and α-SMA, while increasing the epithelial marker E-cadherin. Mechanistic experiments identified an interaction between NEAT1 and miR-204-5p and confirmed Six1 as a direct target of miR-204-5p. The results therefore support a NEAT1/miR-204-5p/Six1 regulatory axis in TGF-β1-induced EMT. Nevertheless, because these experiments were performed in an induced epithelial cell model, confirmation in primary human airway tissue remains necessary.

There is more extensive in vivo evidence linking LincR-PPP2R5C to EMT and airway remodelling. Yuan et al. [105] investigated this lncRNA in a chronic HDM-induced model of allergic asthma. Genetic deficiency of LincR-PPP2R5C attenuates airway remodelling and reverses EMT-associated changes, including reduced E-cadherin and increased N-cadherin and vimentin expression. Mechanistically, LincR-PPP2R5C deficiency increases PP2A activity and reduces epithelial TGF-β1 production. Pharmacological modulation of PP2A further supported a functional relationship between PP2A activity, TGF-β1 expression, and EMT. Notably, the effect of LincR-PPP2R5C deficiency on PP2A activity appears to be cell and context-dependent, as PP2A activity is reduced in differentiating Th2 cells in the acute asthma model [99], whereas it is enhanced in airway epithelial cells in the chronic HDM model [105]. When considered together with its effects on Th2 differentiation in acute allergic inflammation [99], these findings suggest that LincR-PPP2R5C can regulate more than one component of experimental asthma, linking adaptive immune responses with structural airway remodelling.

Overall, the current mechanistic evidence indicates that lncRNAs act at several levels of asthma pathobiology. Lnc-BAZ2B and LincR-PPP2R5C regulate immune cell activation or differentiation [40,99]; CRNDE links activated neutrophils with airway smooth muscle cell responses and NF-κB signalling [100]; MALAT1 and MEG3 modulate airway smooth muscle phenotypes [101,102,103]; and NEAT1 and LincR-PPP2R5C influence EMT-associated epithelial changes [104,105]. These observations support a model in which lncRNAs form a cell- and context-dependent regulatory network connecting airway inflammation with structural remodelling.

The evidence should nevertheless be interpreted with caution. For several representative lncRNAs, including MEG3 and NEAT1, recent mechanistic findings rely predominantly on stimulated cell models rather than direct functional studies in patients with asthma [103,104]. LincR-PPP2R5C is supported by detailed genetic experiments in acute and chronic murine allergic asthma, but its corresponding functional role in human asthma remains to be established [99,105]. MALAT1 is supported by complementary smooth muscle studies, although further validation in primary bronchial tissue from phenotypically defined patient populations is needed [101,102]. Future studies integrating single-cell or spatial transcriptomic approaches with functional validation in primary human cells may help to determine which lncRNA regulatory axes represent central mechanisms of human asthma and which are predominantly model-dependent observations.

## 6. Role of circRNAs in Asthma

### 6.1. circRNA–miRNA–mRNA Regulatory Networks

Most circRNA mechanisms reported in asthma follow the same pattern. A circRNA whose abundance changes in asthmatic tissue binds one or more miRNAs and relieves their repression of a protein-coding target, and that target drives an inflammatory or structural phenotype. More than 20 distinct circRNAs have now been assigned such axes in asthma. Recurring across them are hub circRNAs carrying several axes at once, miRNAs shared between axes, protective as well as pathogenic directionality, mechanisms that do not involve miRNAs, and the transfer of circRNAs between cells. The first asthma-specific functional study appeared in 2019 [106], and since then the field has extended to every major cell type of the airway wall [71].

Certain circRNAs act as hubs. circHIPK3, first characterised as a multi-miRNA sponge in cancer [107], has been assigned two independent axes in airway smooth muscle: sponging of miR-326 to derepress the calcium sensor stromal interaction molecule 1 (STIM1) [108], and sponging of miR-375 to derepress the matrix metalloproteinase 16 (MMP-16) [109]. Both have been studied in PDGF-stimulated cells and have produced the same outcome: increased proliferation and migration alongside suppression of apoptosis. This illustrates a general feature of these networks: convergence on phenotype rather than one-to-one mapping of an axis to an effect. hsa_circ_0002594 shows comparable multiplicity in CD4+ T cells. It is upregulated in asthma, tracks with type 2–high status, and is reduced after inhaled corticosteroid treatment [110]. It promotes Th2 differentiation by binding eukaryotic translation initiation factor 4A3 (eIF4A3) to destabilise PTEN mRNA and activate PI3K/AKT/mTOR signalling [111]. In addition, in airway smooth muscle it sponges miR-139-5p to upregulate tripartite motif-containing 8 (TRIM8) [112]. That the same circRNA operates through a protein-binding mechanism in one cell type and a canonical sponge mechanism in another is a useful corrective to the assumption that competing endogenous (ceRNA) activity is a defining function of circRNAs.

Conversely, individual miRNAs are shared across axes. miR-381-3p is targeted by both circUQCRC2, which derepresses vascular endothelial growth factor A (VEGFA) and activates NF-κB signalling in paediatric asthma [113], and by hsa_circ_0099188, which derepresses protein phosphatase 3 catalytic subunit alpha (PPP3CA) and Krüppel-like factor 4 (KLF4) to enhance NOS2 and chemokine transcription in macrophages [114]. KLF4 is itself a shared endpoint, also reached through hsa_circ_0008726 and miR-206-3p in the same occupational-asthma model [115]. Convergence of several circRNAs on one miRNA is mechanistically significant because the aggregate contribution of a set of co-induced circRNAs can plausibly exceed what any single circRNA could achieve [116].

The directionality of these axes is not uniformly pathogenic. A subset is downregulated in asthma and protective when restored. circ_001372 suppresses inflammatory cytokine production and cyclooxygenase-2 (COX-2)/inducible nitric oxide synthase (iNOS) expression by sponging miR-128-3p to induce SIRT1 [117], and circ_0000029 restrains PDGF-BB-driven smooth muscle proliferation via miR-576-5p/potassium voltage-gated channel subfamily A member 1 (KCNA1) [118]. circ_0070934 inhibits IL-4/IL-13-induced EMT through miR-199a-5p/mannosyl (beta-1,4-)-glycoprotein beta-1,4-N-acetylglucosaminyltransferase (MGAT3) [119], and circ-0001454 alleviates airway inflammation and remodelling via miR-770-5p/Casitas B-lineage lymphoma proto-oncogene B (cbl-b) [120]. circ-0001875 is reduced in severe asthma and normally restrains M1 macrophage polarisation [121], and circDCBLD2 corrects Th1/Th2 imbalance by sponging miR-26a-5p to upregulate phosphatase and tensin homolog (PTEN) [122]. Both signs are therefore represented, which implies that therapeutic strategies need to be axis specific: knockdown for the pathogenic set, restoration for the protective set.

Several of the best-supported asthma circRNA mechanisms are not sponge mechanisms at all. circS100A11, upregulated in children with asthma, competitively binds the RNA-binding protein cell cycle-associated protein 1 (CAPRIN1) to relieve its translational suppression of S100A11. The resulting protein frees SP3 from nucleolin, increasing SP3 occupancy at the STAT6 promoter and driving M2a macrophage activation [123]. Two studies of exercise-mediated protection converge on a related architecture. Epithelial circMETTL9 competes with IGFBP3 for EIF4A3, suppressing IGFBP3 and activating CXCL12–CXCR4 signalling, and aerobic exercise downregulates the circRNA and reverses the phenotype [124,125]. CDR1as, the prototypical miR-7 sponge, acts in asthma through protein axes instead. Transferred to endothelial cells in exosomes from IgE-stimulated mast cells, it signals via fused in sarcoma (FUS) and phosphorylated p65 to upregulate vascular cell adhesion molecule 1 (VCAM-1) and intercellular adhesion molecule 1 (ICAM-1) [126]. In asthma triggered by particulate matter, it binds insulin-like growth factor 2 mRNA-binding protein 2 (IGF2BP2) to promote ferroptosis [127]. Protein sequestration and competition for RNA-binding proteins are thus at least as prominent as miRNA sponging in the mechanistically deepest asthma studies, consistent with the broader circRNA literature [128].

Intercellular transfer adds a further dimension. circEif3c is enriched in exosomes from M2 macrophages and delivered to alveolar epithelial cells, sponging miR-15a-5p to upregulate glutathione synthetase (GSS) and suppressor of cytokine signalling 6 (SOCS6), mitigating airway inflammation and remodelling in ovalbumin-sensitised mice [129]. Together with the mast cell-to-endothelium transfer of CDR1as [126], this indicates that circRNA-mediated regulation in the airway is not confined to the cell of origin. Vesicular delivery may also concentrate a circRNA in a recipient cell to levels at which competitive mechanisms become credible.

### 6.2. Effects on Airway Inflammation and Remodelling

The earliest asthma circRNA study reported a circRNA that is elevated in patient cells, sequesters an miRNA, and thereby increases a type 2 cytokine, a sequence of observations reproduced in most subsequent reports across the immune compartments. hsa_circ_0005519 is elevated in CD4+ T cells and PBMCs of patients with asthma. It binds hsa-let-7a-5p and relieves its suppression of IL-13 and IL-6, increasing the production of both cytokines [106]. Type 2 skewing is the dominant reported consequence of circRNA dysregulation in T cells. hsa_circ_0002594 correlates with type 2–high status as well as disease markers, declines after inhaled corticosteroid therapy, and discriminates patients from controls in receiver-operating-characteristic analysis [110]. Mechanistically, it promotes Th2 differentiation by destabilising PTEN mRNA and activating PI3K/AKT/mTOR signalling, with increased IL-4 and reduced IFN-γ [111]. circDCBLD2 acts in the opposite direction on the same axis. Reduced in CD4+ T cells from patients with asthma, it sponges miR-26a-5p to upregulate PTEN, favouring Th1 over Th2 differentiation, and its restoration alleviates the asthmatic phenotype in ovalbumin-sensitised mice [122]. PTEN is thus a convergence point reached by two circRNAs that produce opposite outcomes through different mechanisms, which strengthens the case for PTEN-dependent PI3K signalling as a genuine node in circRNA control of T cell polarisation.

Macrophage phenotype is the second major inflammatory axis. circS100A11 drives M2a activation and lung inflammation in children with asthma through the CAPRIN1–S100A11–SP3–STAT6 cascade described above [123], whereas circ-0001875 is downregulated in severe asthma and normally restrains M1 polarisation by binding miR-31-5p to permit SP1 translation and consequent NF-κB suppression [124. The two studies address different poles of macrophage activation and different severity strata, and together they indicate that circRNA control of macrophage phenotype is bidirectional rather than uniformly pro-inflammatory. Pharmacological modulation is feasible in principle: luteolin upregulates hsa_circ_0001326 in THP-1-derived macrophages, and this circRNA sponges hsa-miR-136-5p to increase ubiquitin-specific peptidase 4 (USP4), promoting M2 polarisation and suppressing M1 polarisation [130]. Occupational asthma provides the most coherent macrophage subset. Exposure of macrophages to a 4,4′-methylene diphenyl diisocyanate–glutathione conjugate induces hsa_circ_0008726, which sponges miR-206-3p to derepress KLF4 and drive chemokine transcription [115], and hsa_circ_0099188, which targets miR-381-3p to derepress PPP3CA and KLF4 [114]. Two independently identified circRNAs converging on KLF4 in the same exposure model are the clearest example in this literature of multi-circRNA convergence on a shared node.

The epithelium is where circRNA effects map most directly onto recognised asthma pathology. The strongest evidence concerns goblet cell metaplasia: circZNF652 is upregulated in the airway epithelium of children with asthma and in allergen-sensitised mice, where it sponges miR-452-5p to derepress JAK2, activating JAK2/STAT6 signalling [131]. This study combines human paediatric airway epithelial and BALF material with an in vivo model and terminates on a canonical type 2 effector pathway. Allergen-induced epithelial injury is mediated by circVPS33A. It sponges miR-192-5p to derepress the alarmin high mobility group box 1 (HMGB1), promoting Der p1-induced cytokine release and apoptosis while impairing repair-associated migration [132]. circDHTKD1 also increases inflammation: it sponges miR-338-3p to upregulate ETS1 and activate extracellular signal-regulated kinase (ERK) signalling in lipopolysaccharide (LPS)-stimulated bronchial epithelial cells [133,134]. EMT, a mechanism underlying subepithelial fibrosis, is controlled in both directions. circ_0070934 is reduced in asthma and normally inhibits IL-4/IL-13-induced EMT by sponging miR-199a-5p to upregulate MGAT3 [119]. mmu_circ_0000981 acts in the opposite direction: elevated in ovalbumin-sensitised mice, it promotes TGF-β1-driven EMT and pulmonary fibrosis by sponging miR-211-5p to upregulate TGFBR2, terminating on the TGF-β receptor itself [112]. Protective epithelial axes are also described, circ-0001454 acting via miR-770-5p/cbl-b to reduce ROS and apoptosis [120] and exosomal circ-Eif3c protecting alveolar epithelial cells via miR-15a-5p/GSS/SOCS6 [129].

Airway smooth muscle has been studied most intensively, and the published studies have used relatively similar methods. Nearly all studies stimulate primary cells or a cell line with PDGF; manipulate the circRNA; and examine proliferation, migration, and apoptosis as the outcomes. Seven axes have been reported: circHIPK3 through miR-326/STIM1 [108] and miR-375/MMP-16 [109]; circERBB2 through miR-98-5p/IGF1R [135]; circ_0002594 through miR-139-5p/TRIM8 [112]; circ_CSNK1E through miR-34a-5p/vesicle-associated membrane protein 2 (VAMP2) [136,137,138]; circUQCRC2 through miR-381-3p/VEGFA with downstream NF-κB activation [113]; and the single protective axis, circ_0000029, which restrains proliferation and migration while promoting apoptosis via miR-576-5p/KCNA1 [118]. The downstream effectors are mechanistically diverse yet functionally coherent: a store-operated calcium entry sensor (STIM1), a matrix metalloproteinase (MMP-16), IGF1R, an E3 ligase component (TRIM8), a vesicle fusion protein (VAMP2), an angiogenic factor (VEGFA), and a potassium channel (KCNA1). The recognised substrates of smooth muscle hyperplasia and hypercontractility that constitute remodelling—calcium handling, matrix turnover, growth factor signalling, and contractile function—are all represented [10]. The fact that seven independent manipulations converge on the same proliferative and migratory phenotype through distinct effectors suggests that rather than acting through a single bottleneck, circRNAs are integrated into smooth muscle growth control. The uniformity of the experimental design is nonetheless a limitation. PDGF stimulation of cultured smooth muscle cells primarily models proliferative activation rather than asthmatic airway remodelling. Moreover, interventional evidence in this compartment is limited to in vitro studies; no study has yet demonstrated that manipulating a smooth muscle circRNA alters airway wall structure or airway hyperresponsiveness in vivo (Figure 3).

## 7. Crosstalk Between Non-Coding RNAs and Signaling Pathways

lncRNAs are increasingly recognised as modulators of NF-κB signalling in inflammatory lung disorders. The available evidence suggests that they can influence NF-κB activity through effects on upstream regulatory molecules, inhibitory mechanisms, and downstream inflammatory gene expression. Disrupted lncRNA–NF-κB interactions may contribute to persistent inflammation and lung remodelling, while targeting this regulatory network may offer future therapeutic opportunities [139].

NF-κB is a central regulator of airway inflammation. Its activation in epithelial cells, macrophages, mast cells, and airway smooth muscle cells induces the expression of pro-inflammatory cytokines, chemokines, and adhesion molecules, including TNF-α, IL-1β, IL-6, and IL-8 [140]. These factors promote inflammatory cell infiltration and contribute to airway hyperresponsiveness. ncRNAs can regulate NF-κB signalling at multiple stages, including receptor-associated signalling events, the functions of inhibitory proteins, and the transcription of NF-κB-dependent inflammatory targets. In asthma-associated mast cell inflammation, the lncRNA AK149641 interacts with NF-κB within the nucleus. Downregulation of AK149641 is accompanied by reduced nuclear NF-κB DNA-binding activity and weaker lipopolysaccharide-induced inflammatory responses in mast cells [141,142].

In airway smooth muscle cells, taurine upregulated 1 (TUG1) may act as a ceRNA by sequestering miR-181b, thereby increasing HMGB1 expression and promoting airway remodelling via an NF-κB-related pathway. In addition, miR-221 has been reported to increase mast cell IL-4 production through modulation of PTEN and p38/NF-κB signalling, which may facilitate Th2-cell polarisation. Zhang et al. [143] reported significant upregulation of miR-221 in bronchial epithelial cells isolated from patients with asthma, accompanied by a reduction in SIRT1 mRNA expression. Furthermore, miR-221 levels are inversely correlated with SIRT1 mRNA abundance in patient-derived samples, supporting a potential regulatory relationship between miR-221 and SIRT1 in the asthmatic airway epithelium.

The JAK/STAT cascade is essential for cytokine signalling in asthma. IL-4 and IL-13 activate STAT6, promoting type 2 inflammation, IgE-associated responses, mucus hypersecretion, and alternative macrophage activation. IL-5-related pathways support eosinophil survival and activity, while STAT3 and STAT5 participate in epithelial repair, inflammatory signalling, and lymphocyte differentiation. Aberrant ncRNA expression can therefore alter both T2-high and non-T2 inflammatory endotypes by regulating cytokine JAK/STAT signal transduction.

Another example is the lncRNA NEAT1/miR-139/JAK3/STAT5 axis. In airway smooth muscle cells obtained from asthma-associated models, increased NEAT1 expression accompanied by reduced miR-139 levels has been linked to JAK3/STAT5 pathway activation and enhanced production of the pro-inflammatory mediators TNF-α, IL-1β, IL-6, and IL-8. NEAT1 overexpression upregulates the expression of TNF-α, IL-1β, IL-6, and IL-8. In contrast, overexpressing miR-139 or inhibiting the JAK3/STAT5 pathway attenuates these NEAT1-induced pro-inflammatory effects [144].

In macrophages, miR-142-5p may contribute to sustained cytokine signalling by targeting SOCS1, a key negative regulator of this pathway. SOCS1 inhibition may prolong STAT6 phosphorylation, thereby facilitating M2 macrophage polarisation and promoting profibrotic activity [145].

Researchers have investigated the role of miR-130a-3p in experimental models of pulmonary fibrosis [146]. They reported that its activity is associated with both inflammatory and fibrotic stages of disease progression. However, the key mechanisms identified in this study appear to extend beyond the modulation of the peroxisome proliferator-activated receptor gamma (PPARγ)/STAT6 macrophage signalling pathway.

In a PDGF-BB-induced human airway smooth muscle cell model of asthma-associated airway remodelling, lncRNA MEG3 is a regulator of cellular proliferation and migration through the miR-143-3p/FGF9 axis. MEG3 expression is inversely associated with miR-143-3p, whereas miR-143-3p directly targets and negatively regulates FGF9. Silencing MEG3 increases miR-143-3p expression and consequently reduces FGF9 levels, leading to decreased human airway smooth muscle cell proliferation, proliferating cell nuclear antigen (PCNA) expression, and migratory capacity. These findings suggest that the MEG3/miR-143-3p/FGF9 regulatory network contributes to PDGF-BB–driven airway smooth muscle remodelling and may represent a potential therapeutic target in asthma [103].

ncRNAs may modulate JAK/STAT pathway activity by regulating negative feedback mediators, including SOCS proteins, or by altering transcription elicited by cytokine stimulation. This regulatory capacity is especially pertinent to airway remodelling, in which alternatively activated macrophages and STAT6-driven signalling link type 2 inflammation to fibrotic processes and excessive ECM deposition. miR-378 is upregulated in blood and lung samples from children with asthma and may promote airway smooth muscle cell proliferation, migration, ECM production, and resistance to apoptosis. Its predicted targets are enriched in erythroblastic leukaemia viral oncogene homolog (ErbB), Ras, MAPK, and calcium-signalling pathways, supporting a role in airway remodelling. In immune cells, the lncRNA PTPRE-AS1 restrains IL-4-induced M2 macrophage activation by increasing PTPRE expression and suppressing ERK1/2 signalling. PTPRE-AS1 deficiency enhances M2-associated responses and aggravates allergic pulmonary inflammation, suggesting potential relevance to type 2 asthma [147].

In airway epithelial cells, miR-200a and miR-200b have been reported to suppress inflammatory signalling by directly targeting ORMDL3, an asthma-susceptibility gene. Inhibition of either miRNA increases ORMDL3 expression, ERK phosphorylation, MMP-9 abundance, and the production of TNF-α, IL-4, IL-5, IL-13, and IL-1β in A549 epithelial cells. These observations support a miR-200a/miR-200b–ORMDL3–ERK/MMP-9 regulatory axis that may link epithelial inflammatory activation with remodelling-associated protease activity [148].

Several lncRNA–miRNA regulatory circuits converge on the PTEN/PI3K/Akt axis, a key pathway implicated in airway smooth muscle cell proliferation, migration, airway remodelling, and corticosteroid responsiveness. In airway smooth muscle cells derived from patients with severe asthma, the lncRNA CASC7 is downregulated, whereas miR-21 expression and Akt activity are increased. CASC7 acts as a ceRNA for miR-21, thereby relieving miR-21-mediated repression of PTEN. Restoration of PTEN expression inhibits PI3K/Akt signalling and enhances the inhibitory effects of dexamethasone on airway smooth muscle cell proliferation and inflammatory cytokine secretion. These findings identify CASC7 loss as a potential contributor to corticosteroid-insensitive airway remodelling [149].

The lncRNA H19 represents another regulator of this axis. H19 expression is decreased in serum from children with asthma and in PDGF-BB-stimulated airway smooth muscle cells, while miR-21 is increased. In vitro, H19 overexpression sequesters miR-21, restores PTEN protein expression, reduces Akt phosphorylation, and suppresses PDGF-BB-induced airway smooth muscle cell proliferation and migration. The reversal of these effects by miR-21 overexpression or PTEN silencing provides mechanistic evidence that H19 can restrain remodelling-associated airway smooth muscle cell responses through the miR-21/PTEN/Akt cascade [150].

In severe asthma, reduced epithelial miR-744 expression has been associated with increased bronchial epithelial-cell proliferation. Studies indicate that miR-744 directly targets TGF-β1, reducing TGF-β1 expression and Smad3 phosphorylation while increasing Smad anchor for receptor activation (SARA) abundance. These changes suppress epithelial-cell proliferation [151].

In airway smooth muscle cells, miR-204-5p is downregulated following TGF-β1 stimulation and in asthma-derived cells. Restoration of miR-204-5p limits TGF-β1-induced proliferation and the deposition of fibronectin and collagen III through direct repression of SIX1 [152].

The lncRNA RMRP may further contribute to asthma-related remodelling through a miR-206/CCL2-dependent mechanism. In experimental asthma, increased RMRP sequesters miR-206 and increases CCL2 expression, with downstream enhancement of inflammatory cytokine production and airway smooth muscle cell viability, and inhibition of apoptosis. Pharmacological inhibition of TGF-β/Smad2 signalling attenuates these effects, indicating that RMRP acts upstream of, or in concert with, TGF-β/Smad2 signalling [153].

TGF-β signalling is embedded within a broader profibrotic network rather than functioning in isolation. It intersects with the PI3K/Akt, MAPK, NF-κB, β-catenin, and type 2 cytokine pathways, thereby linking inflammatory stimuli to persistent airway wall remodelling. Accordingly, ncRNAs that regulate the TGF-β axis may influence both inflammatory activity and the structural abnormalities characteristic of chronic asthma.

miR-223 is an important negative regulator of the NLR family pyrin domain containing 3 (NLRP3) inflammasome pathway in neutrophilic airway inflammation. By directly targeting NLRP3, miR-223 limits inflammasome availability and thereby restrains caspase-1-dependent IL-1β production. In experimental neutrophilic asthma, miR-223 deficiency exacerbates airway inflammatory cell infiltration, inflammatory cytokine release, NLRP3 inflammasome activity, and IL-1β production, whereas miR-223 replacement attenuates these responses. These findings indicate that impaired miR-223-mediated repression of NLRP3 may lower the threshold for inflammasome activation and promote IL-1β-driven neutrophilic airway inflammation [154].

NF-κB contributes to the priming phase of inflammasome activation by inducing transcription of NLRP3 and IL1B, thereby increasing the intracellular availability of NLRP3 and pro-IL-1β. miR-223 provides a post-transcriptional counter-regulatory mechanism by limiting NLRP3 abundance. Accordingly, an imbalance between NF-κB-mediated inflammasome priming and miR-223-dependent inhibition may increase NLRP3 activation and intensify IL-1β-driven inflammation in neutrophilic asthma. In an ovalbumin-induced rat model, bone marrow mesenchymal stem cell–derived exosomes carrying miR-223-3p reduce airway inflammation and remodelling, concomitantly with inhibition of the NLRP3/apoptosis-associated speck-like protein containing a CARD (ASC)/caspase-1/gasdermin D (GSDMD) pathway [155].

The relationship between ncRNAs and the Keap1–Nrf2 antioxidant pathway is an emerging area of investigation in asthma. Nrf2 is normally restrained by its redox-sensitive repressor, Keap1, but oxidative or electrophilic stress disrupts this interaction and allows Nrf2 to accumulate in the nucleus. Nrf2 subsequently induces antioxidant and cytoprotective genes, including HMOX1, NQO1, glutathione-related enzymes and other components of the cellular defence system [156].

A mechanistic model is provided by miR-200a, which directly targets KEAP1 mRNA. Reduced miR-200a can increase Keap1 expression, diminish Nrf2-dependent antioxidant activity, and permit ROS accumulation. In a fructose-induced hepatic inflammation model, this sequence is associated with thioredoxin-interacting protein (TXNIP) induction, NLRP3 inflammasome activation, and increased IL-1β production. Thus, a miR-200a/Keap1/Nrf2/ROS/TXNIP/NLRP3 cascade provides a biologically plausible framework through which ncRNA dysregulation could couple oxidative stress to inflammasome activation in asthma [157].

The available asthma evidence nevertheless supports a protective role for Nrf2 signalling. In a recent allergen-induced murine asthma study, the authors reported that Nrf2 deficiency increases oxidative stress and exacerbates neutrophilic airway inflammation, with increased Th17-associated cytokine production and RAR-related orphan receptor gamma t (RORγt) expression. These findings suggest that impaired Nrf2-dependent antioxidant defence may favour a neutrophilic/Th17 inflammatory environment. Therefore, aberrant ncRNA regulation of the Keap1–Nrf2 system could potentially influence oxidative injury, airway inflammation, and inflammasome susceptibility, but direct asthma-specific validation of ncRNA–Nrf2 mechanisms is required.

## 8. Clinical Applications of ncRNAs in Asthma

Asthma is clinically and biologically heterogeneous, and the currently available biomarkers do not fully capture the complexity of individual inflammatory endotypes, airway remodelling processes, or future disease risk. ncRNAs may complement conventional measures such as blood eosinophil count, fractional exhaled nitric oxide, serum IgE, spirometry, and clinical assessment by reflecting regulatory networks involved in type 2 inflammation, neutrophilic inflammation, epithelial dysfunction, and structural airway changes. lncRNAs may offer additional diagnostic and prognostic information because of their close involvement in transcriptional regulation, chromatin remodelling, cytokine signalling, and ceRNA networks. For example, the lncRNA NEAT1 has been associated with asthma activity, inflammatory burden, impaired lung function, and exacerbation risk. In one clinical cohort, researchers reported higher circulating NEAT1 levels during exacerbation than during remission or in healthy controls, and an inverse correlation between NEAT1 expression and miR-124 expression.

Similarly, the ANRIL/miR-125a regulatory axis has been proposed as a potential indicator of disease deterioration, severity, and inflammation [158,159]. Several circulating miRNAs, including miR-155, let-7a, miR-21, miR-98, and members of the let-7 family, have been associated with greater asthma severity and eosinophilic phenotypes. ncRNAs may also support exacerbation prediction. Circulating miR-146b, miR-206, and miR-720 have been proposed as candidate biomarkers of future exacerbations in children with asthma [160]. Increased NEAT1 expression has similarly been linked to more frequent and severe exacerbations, elevated pro-inflammatory cytokine levels, and poorer lung function parameters. In addition, ncRNAs may help identify corticosteroid responsiveness. Circulating miR-155-5p and miR-532-5p have been associated with longitudinal responses to inhaled corticosteroids and dexamethasone-mediated NF-κB repression [161]. Increased PVT1 expression in airway smooth muscle cells has also been linked to severe asthma and corticosteroid resistance.

Liquid biopsy involves the analysis of molecular biomarkers in accessible biological specimens. In asthma, circulating ncRNAs are attractive candidates because they can be repeatedly sampled and may enable longitudinal assessment of inflammatory activity without bronchoscopy. miRNAs can be detected in plasma or serum either bound to proteins or encapsulated in EVs, including exosomes. Vesicular packaging may protect RNA molecules from degradation and may partly reflect the cellular source of inflammatory signalling. Eosinophil-derived EV ncRNAs are therefore of potential interest for the non-invasive evaluation of eosinophilic asthma. In addition, blood-based detection of lncRNAs, such as NEAT1, and selected circRNAs expands the range of potential liquid-biopsy biomarkers [158].

However, substantial methodological challenges remain. ncRNAs are also potential therapeutic targets because they regulate multiple pathways relevant to asthma pathogenesis. Approaches may involve replacement of protective miRNAs with synthetic mimics; inhibition of pathogenic miRNAs using anti-miRs, antagomirs, or locked nucleic acid inhibitors; and lncRNA suppression with antisense oligonucleotides, small interfering RNAs, or CRISPR-based transcriptional modulation. Disease-promoting circRNAs could potentially be inhibited using oligonucleotides targeting back-splice junctions, whereas engineered circRNAs may serve as stable miRNA sponges or RNA-delivery platforms. PVT1-related pathways may represent targets in airway remodelling and corticosteroid resistance, whereas NEAT1-associated networks may influence inflammatory activation and exacerbation risk. Nevertheless, evidence for ncRNA-directed treatment in human asthma remains insufficient, and these strategies should currently be regarded as preclinical [158,162]. Lipid, polymeric, inorganic, and EV-inspired nanoparticles may improve RNA stability and intracellular delivery. Inhaled formulations are particularly promising because they could increase local lung exposure while reducing systemic distribution. Figure 4 summarizes the major nanoparticle- and inhalation-based delivery strategies for therapeutic ncRNAs and ncRNA-targeting molecules discussed below, together with their principal advantages and limitations. Translation is complicated by asthma heterogeneity, the pleiotropic effects of ncRNAs, inconsistent findings from small cross-sectional studies, and the difficulty of targeting specific airway cell populations [163]. Manufacturing reproducibility, biodistribution, immunogenicity, long-term safety, dosing, and regulatory requirements require further evaluation.

These observations suggest that ncRNA-mediated regulation of PTEN and Akt may connect airway remodelling with impaired corticosteroid responsiveness. Strategies that restore PTEN activity by inhibiting miR-21 or restoring CASC7/H19 expression could theoretically reduce airway smooth muscle cell hyperplasia and improve glucocorticoid responsiveness [164]. However, the available studies are largely based on cultured airway smooth muscle cells, PDGF-BB stimulation, and relatively small clinical sample sets. Robust validation in well-phenotyped patient cohorts, including severe asthma and steroid-resistant endotypes, as well as in vivo target engagement and safety studies, is still needed [164]. Overall, the most immediate clinical application of ncRNAs is likely to be standardised and externally validated circulating biomarker panels used alongside clinical, functional, and inflammatory measures. In contrast, inhaled nanoparticle-mediated ncRNA therapeutics remain a promising but early-stage approach that requires rigorous mechanistic, pharmacokinetic, toxicological, and randomised clinical investigation before integration into asthma care.

## 9. Discussion and Future Perspectives

The evidence summarized in this review highlights the important role of ncRNAs in the complex pathogenesis of asthma. miRNAs, lncRNAs, and circRNAs participate in the regulation of immune responses, airway inflammation, epithelial dysfunction, oxidative stress, and airway remodeling, acting through major signaling pathways including NF-κB, JAK/STAT, MAPK, PI3K/Akt, TGF-β/Smad, and NLRP3. Among these, miRNAs remain the most extensively studied class, with molecules such as miR-21, miR-155, miR-146a, miR-126, and miR-223 implicated in inflammatory responses and airway remodeling; emerging evidence also supports roles for lncRNAs including NEAT1, MALAT1, MEG3, H19, and PVT1, whereas circRNAs remain a promising but still relatively underexplored area of asthma research. Beyond their mechanistic contribution to disease, ncRNAs may hold clinical value as biomarkers of disease severity, exacerbation risk, and treatment response.

Translation into clinical practice remains limited by several factors: many studies rely on small and heterogeneous cohorts, differences in sample type, RNA isolation, normalization, and analytical approach complicate cross-study comparison, and altered expression does not, by itself, establish a causal role, underscoring the need for further functional validation. Addressing these gaps will require large, well-characterized, longitudinal cohorts; standardized ncRNA detection and analysis; and integration of ncRNA profiles with genomic, transcriptomic, and proteomic data to refine asthma phenotyping and support precision medicine. Advances in nanoparticle-based and inhaled delivery systems (Figure 4) may in turn enable ncRNA-targeted therapies, provided that issues of specificity, safety, and off-target effects are resolved.

Overall, ncRNAs represent an important regulatory layer in asthma pathogenesis and promising candidates for biomarker development and targeted therapy. Nevertheless, further mechanistic, translational and clinical studies are required before ncRNA-based approaches can be incorporated into personalized asthma management. This regulatory layer captures much of what makes asthma biologically heterogeneous, and its continued characterisation is likely to inform both future biomarker panels and the next generation of precision therapeutics for the disease.

## Figures and Tables

**Figure 1:**
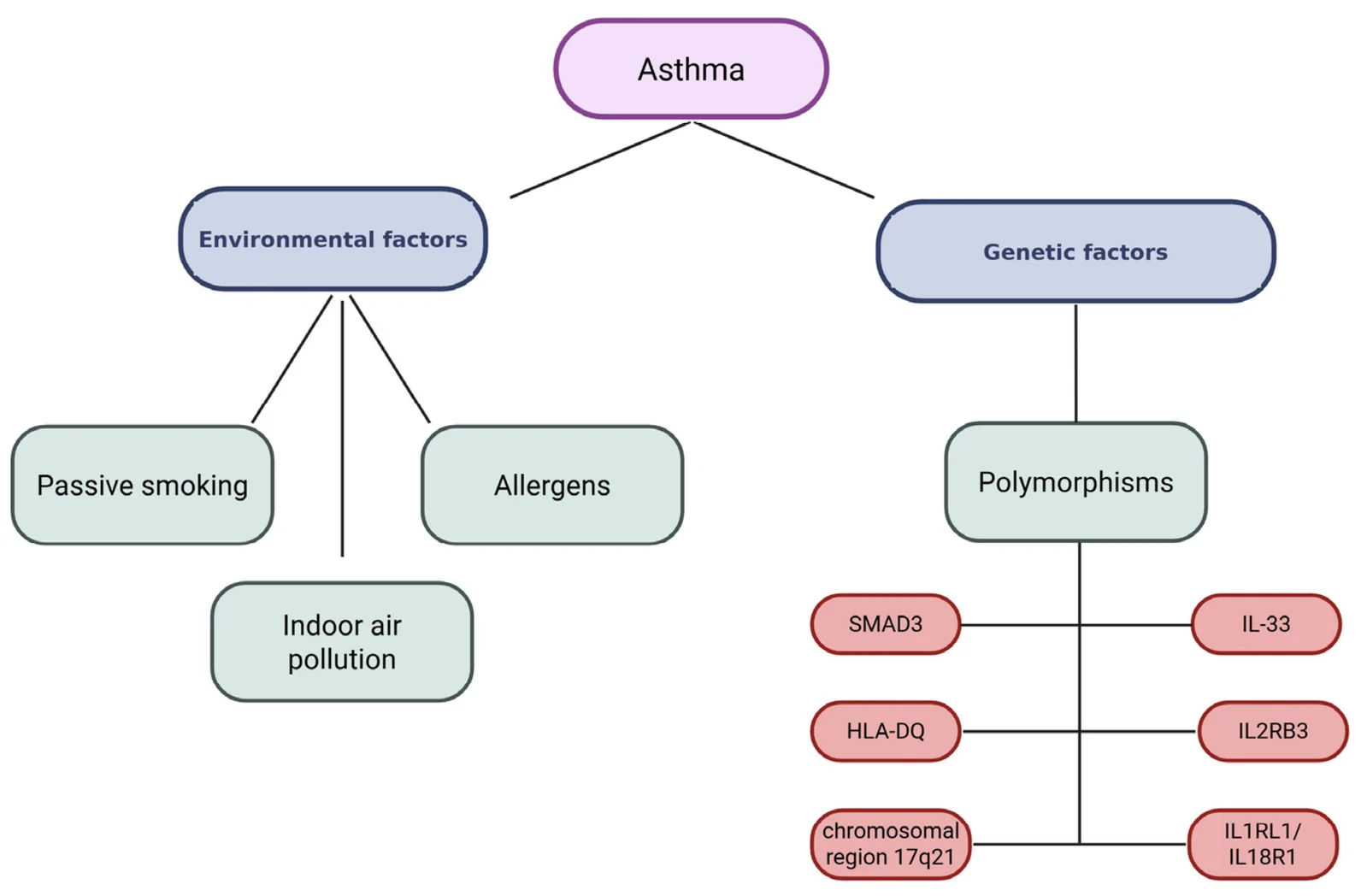
Genetic and environmental factors contributing to the development of asthma. The development of atopic diseases, including asthma, results from complex interactions between genetic susceptibility and environmental exposures. Created in BioRender. Plewa, P. (2026) https://BioRender.com/lvbkoch.

**Figure 2:**
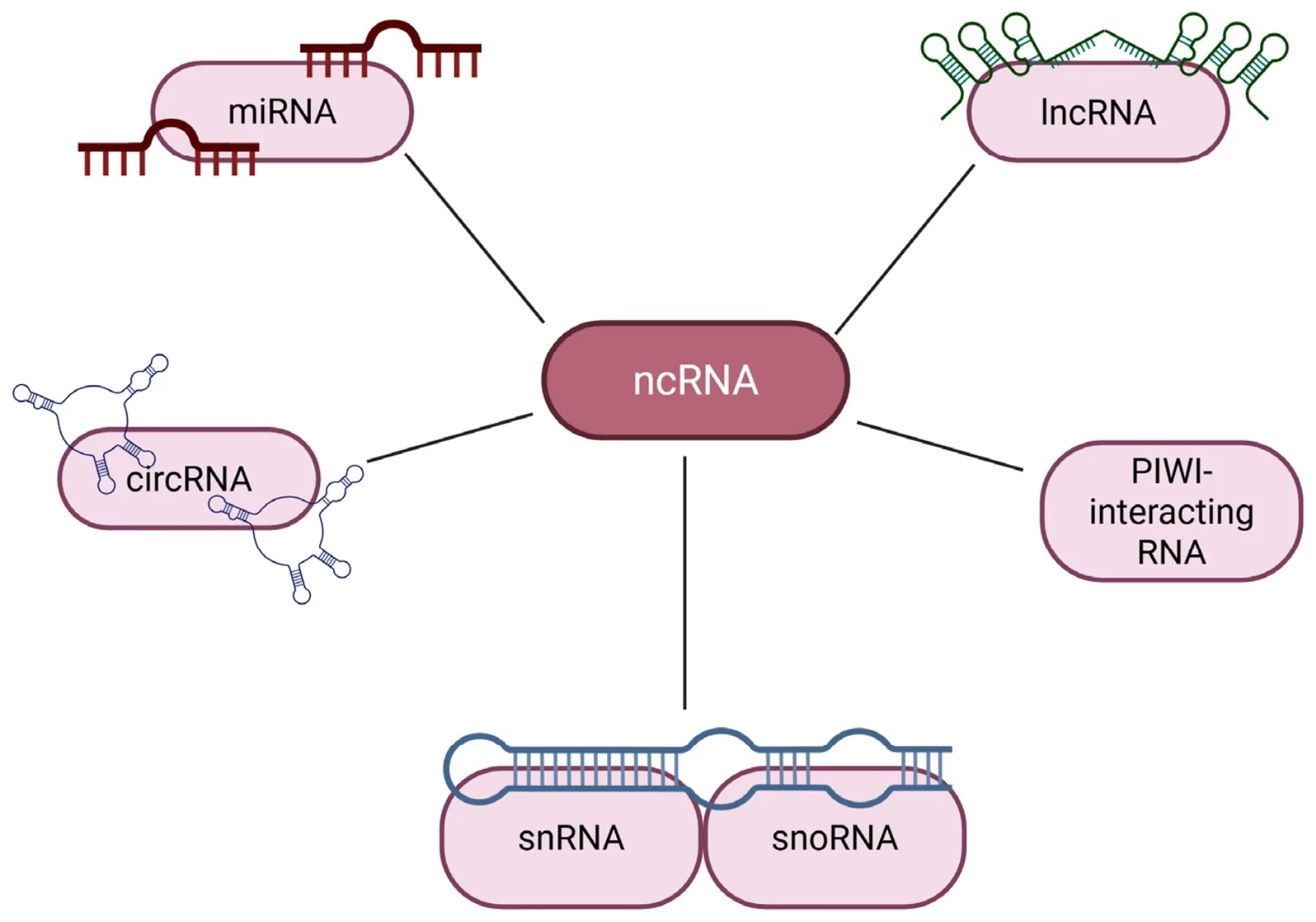
Types of ncRNA. NcRNAs comprise diverse RNA molecules that regulate gene expression at transcriptional, post-transcriptional, and epigenetic levels. The major classes include miRNAs, lncRNAs, circRNAs, snRNAs, snoRNAs, and piRNAs. These ncRNAs interact with specific molecular targets, including messenger RNAs, proteins, chromatin-associated complexes, and other RNA molecules, thereby modulating gene expression, RNA stability, splicing, translation, and epigenetic processes. Dysregulation of ncRNA expression and activity may influence key biological processes involved in asthma pathogenesis, including airway inflammation, immune-cell activation, airway epithelial dysfunction, mucus production, and airway remodeling. Created in BioRender. Plewa, P. (2026) https://BioRender.com/2b7msc4.

**Figure 3:**
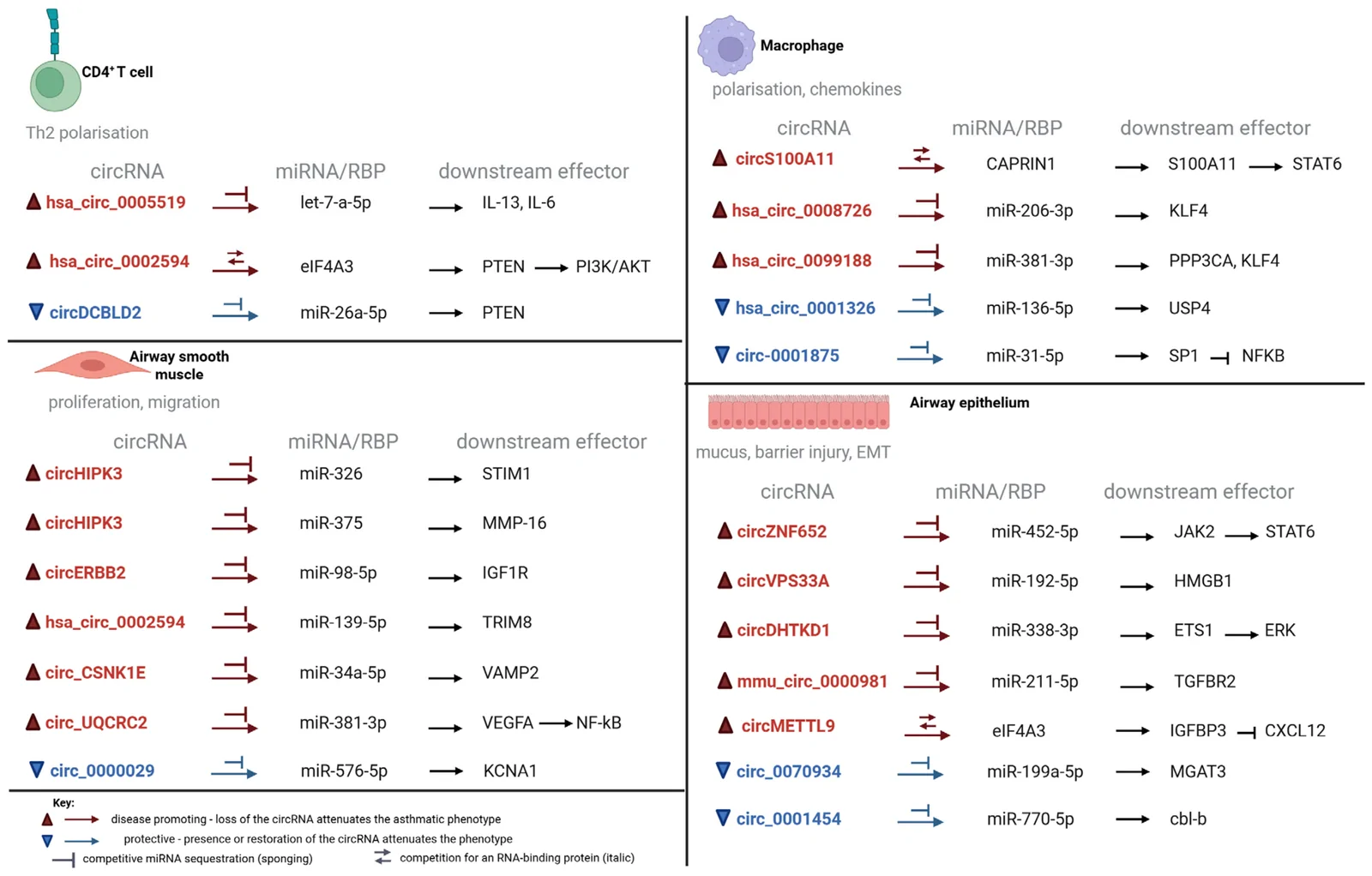
Summarisation of the experimentally reported axes by airway cell compartment. Read as a whole, the evidence supports three conclusions. Reported circRNA dysregulation in asthma is bidirectional, with pathogenic and protective species in every compartment examined. The axes converge on a limited number of well-established downstream pathways—JAK2/STAT6, PI3K/AKT/PTEN, NF-κB, TGF-β/TGFBR2 and growth factor receptor signalling -rather than revealing wholly new effector routes, which increases confidence that the observations are biologically real while tempering claims of mechanistic novelty. And the mechanisms are heterogeneous, with protein sequestration and RNA-binding-protein competition prominent alongside miRNA sponging. Created in BioRender. Birger, R. (2026) https://BioRender.com/k9cipfa.

**Figure 4:**
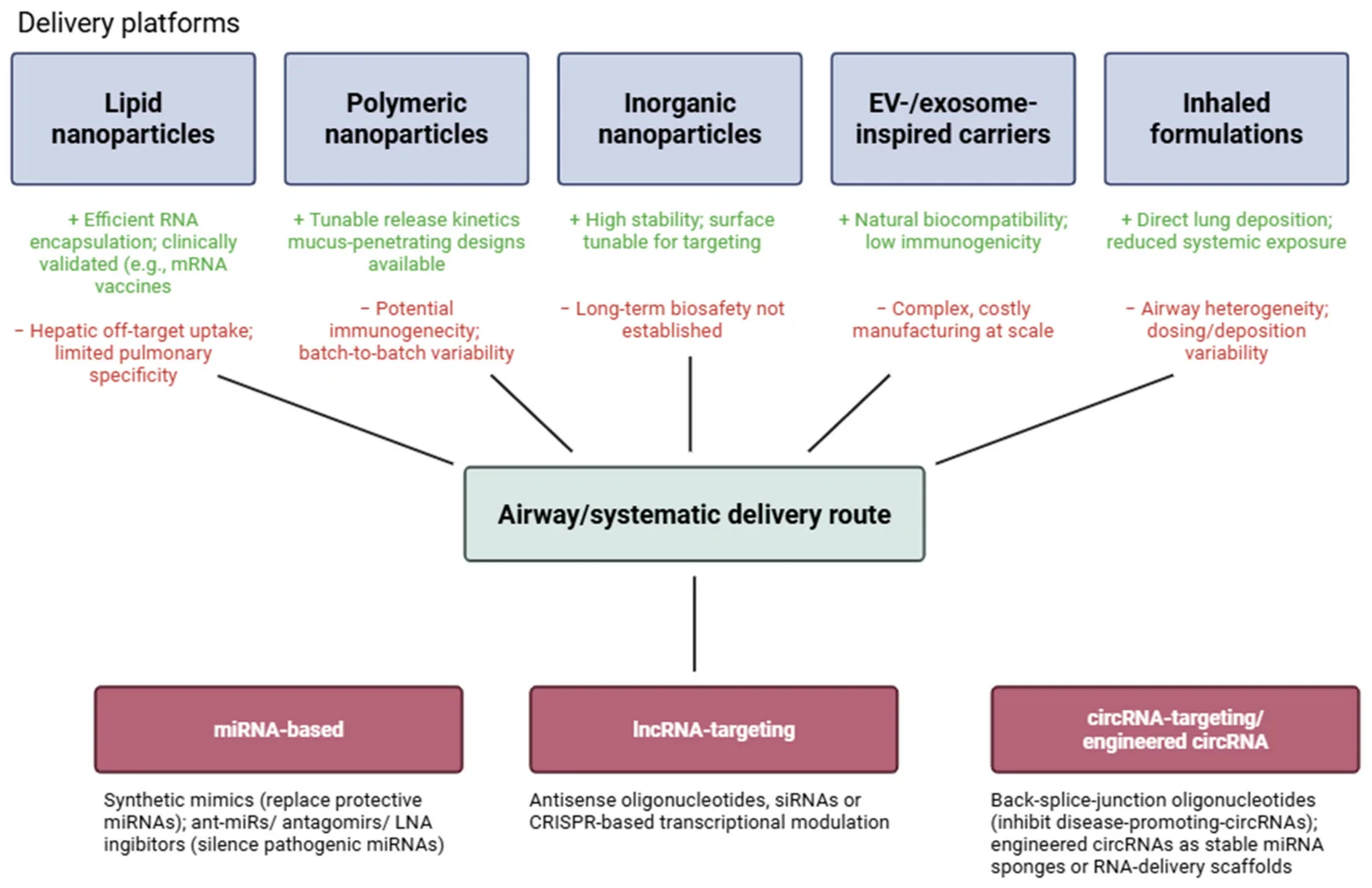
Overview of delivery strategies for therapeutic ncRNAs and ncRNA-targeting molecules in asthma. Nanoparticle- and inhalation-based platforms deliver miRNA-, lncRNA-, and circRNA-targeting cargoes to the airway/systemic compartments; principal advantages (+) and limitations (−) of each platform are indicated.

**Table 1:** Design and principal findings of studies reporting ncRNA dysregulation in asthma discussed in this section.

Cohort/Sample Size	Biological Sample	Platform/Method	Comparison Groups	Title Principal ncRNA Findings	References
4 asthma vs. 3 healthy (GEO GSE195599)	Peripheral whole blood	RNA-seq (Illu-mina HiSeq 2500) + RT-qPCR validation	Asthma vs. healthy controls	1137 DE lncRNAs (485 ↑/652 ↓)	[29]
Children with asthma vs. age-matched controls	PBMCs	RNA-seq + functional follow-up	Asthma vs. healthy controls	719 DE lncRNAs; lnc-BAZ2B enriched in monocytes, mechanistically characterised	[52]
Children with asthma	PBMCs	High-throughput sequencing + validation cohort	Asthma vs. healthy controls	miR-31-5p altered; not all sequencing signals replicated on validation	[53]
361 children (whole-blood); independent adult cohort	Whole blood	Small RNA-seq	Lower vs. higher airflow (FEV1 threshold)	Let-7e-5p ↑, miR-342-3p ↓ with lower airflow; concordant direction in adults	[54]
Asthma vs. healthy controls	Bronchial biopsies	miRNA profiling, adjusted for smoking/ICS	Asthma vs. healthy controls	Distinct miRNA patterns in remission; miRNAs and lncRNAs both prominent in remission network	[55]
Complete remission vs. persistent asthma vs. healthy	Bronchial biopsies	miRNA profiling + integrated network analysis	Remission vs. persistent vs. healthy	Distinct miRNA patterns in remission; miRNAs and lncRNAs both prominent in remission network	[56]
Severe asthma, stratified by OCS exposure	Serum and lung tissue	Next-generation sequencing + validation	OCS-treated vs. non-OCS severe asthma	11 DE serum miRNAs (5 validated); non-identical lung-tissue miRNA set	[57]
Children stratified by lung function	Plasma and saliva (exosomal)	Exosomal ncRNA profiling	Severe airflow obstruction vs. normal lung function	27 DE plasma/40 DE saliva miRNAs; only 5 shared between compartments	[58]
Healthy vs. severe T2-high vs. T2-low asthma	Plasma and induced sputum (exosomal)	Small RNA-seq + independent-cohort validation	T2-high vs. T2-low vs. healthy	Distinct exosomal miRNA profiles by inflammatory endotype in both compartments	[59]
Severe asthma vs. healthy controls	Bronchial epithelial cells	tsRNA + mRNA integrated profiling	Severe asthma vs. healthy controls	58 DE tsRNAs, 961 DE mRNAs; 343 tsRNA-mRNA regulatory pairs, 36 hub mRNAs validated	[60]
No asthma vs. mild vs. severe asthma	Serum	Candidate miRNA panel (35 miRNAs)	Mild vs. severe asthma	Severe asthma: miR-223-3p ↑, miR-30a-5p ↓, miR-660-5p ↓, miR-125b-5p ↓	[61]
Children with asthma	Peripheral blood (circulating piRNAs)	piRNA profiling	T2-high vs. other phenotypes	piRNA signature associated with eosinophil count, total IgE, and T2-high phenotype	[62]

**Table 2:** Key asthma-associated miRNAs: expression patterns, molecular targets, and biological effects.

miRNA	Expression in Asthma	Molecular Targets/Pathways	Major Biological Effects in Asthma	References
miR-21	↑ bronchial epithelium/plasma; particularly increased in eosinophilic asthma	Suppression of IL-12; regulation of TGF-β/Smad pathway through targeting Smad7/TGFβRII	Promotes Th2 polarization, eosinophilia, airway remodeling and fibrosis	[64,86,87,89,90]
miR-126	↑ bronchial epithelial cells; associated with allergic/Th2 asthma	Regulation of IL-5 and IL-13; indirect enhancement of GATA-3 expression	Promotes Th2 inflammation, eosinophilic inflammation, mucus production and AHR; inhibition reduces eosinophil and neutrophil recruitment	[63,64,72,89]
miR-146a	↑ circulation; ↓ airway epithelium/bronchial tissue; compartment-dependent	Targets IRAK1/TRAF6; inhibits TLR/NF-κB and IL-1 signaling	Negative regulation of inflammatory signaling; protects bronchial epithelial cells from cytokine-induced apoptosis; dysregulated expression may contribute to persistent inflammation	[64,84,91,92,93]
miR-155	↑ plasma/airway epithelium; ↓ exhaled breath condensate; compartment-dependent	Regulation of STAT3/STAT5, SOCS1/JAK-STAT, and Jarid2/PRC2 pathways	Modulates Th1/Th2 responses and Th17/Treg balance; promotes Th17 differentiation/function; may promote bronchial smooth muscle proliferation and migration	[73,74,75,76,77,94,95,96]
Let-7 family	↓ airway epithelium, bronchial biopsies, serum/plasma; particularly reduced in severe asthma	Direct targeting of IL-13	Suppresses IL-13, allergic inflammation, mucus metaplasia and subepithelial fibrosis	[77,97,98]
miR-1248	circulating/PBMC-associated findings	Interaction with the IL-5 3′UTR	Enhances IL-5 expression and may promote eosinophil survival, differentiation, recruitment and activation	[76]
miR-223-3p	enriched/highly expressed in airway neutrophils	Associated with TLR signaling, IL-17 responses and ER stress-related gene networks	Associated with airway neutrophilia and severe asthma	[82]
miR-106a	↑ lung tissue, in experimental asthma models	Regulation of IL-10 expression	Knockdown reduces airway inflammation, goblet cell metaplasia and subepithelial fibrosis while increasing IL-10	[63,81,99]
miR-133a	↓ asthmatic airway smooth muscle cells	Direct targeting of IGF1R → PI3K/AKT/mTOR inhibition	Reduces airway smooth muscle activation, α-SMA expression, airway wall thickening and smooth muscle hypertrophy	[63,98]

## Data Availability

No new data were created or analyzed in this study. Data sharing is not applicable to this article.

## Abbreviations

The following abbreviations are used in this manuscript:

ASMC	Airway smooth muscle cell
AP-1	Activator protein-1
BALF	Bronchoalveolar lavage fluid
CAT	Catalase
circRNA	Circular RNA
CRNDE	Colorectal neoplasia differentially expressed
ECM	Extracellular matrix
EMT	Epithelial–mesenchymal transition
EV	Extracellular vesicle
FGF9	Fibroblast growth factor 9
GPX	Glutathione peroxidase
GWAS	Genome-wide association studies
HASMC	Human airway smooth muscle cell
IGF1R	Insulin-like growth factor 1 receptor
IKKβ	Inhibitor of nuclear factor kappa-B kinase β
KCNA	Potassium channel
LA	Linoleic acid
lncRNA	Long Non-Coding RNA
MALAT1	Metastasis-associated lung adenocarcinoma transcript 1
MEG3	Maternally expressed gene 3
MMP	Matrix metalloproteinase
NEAT1	Nuclear paraspeckle assembly transcript 1
miRNA	MicroRNA
ncRNA	Non-coding RNA
NF-κB	Nuclear factor- κB
NO_2_	Nitrogen dioxide
PDGF-BB	Platelet-derived growth factor-BB
piRNA	PIWI-interacting RNA
PM_2_._5_	Particulate matter
PBMC	Peripheral blood mononuclear cells
RNS	Nitrogen species
ROS	Reactive oxygen
SHS	Secondhand smoke
SOCS1	Suppressor of cytokine signaling 1
SOD	Superoxide dismutase
STIM1	Store-operated calcium entry sensor 1
TGF-β1	Transforming growth factor-β1
Th2	T helper 2 lymphocytes
TLR	Toll-like receptor
tsRNA	Transfer RNA-derived small RNA
VAMP	Vesicle fusion protein
VEGFA	Angiogenic factor
